# Persistence of an intact HIV reservoir in phenotypically naive T cells

**DOI:** 10.1172/jci.insight.133157

**Published:** 2020-10-15

**Authors:** Emmanuele Venanzi Rullo, Marilia Rita Pinzone, LaMont Cannon, Sam Weissman, Manuela Ceccarelli, Ryan Zurakowski, Giuseppe Nunnari, Una O’Doherty

**Affiliations:** 1 Department of Pathology and Laboratory Medicine, University of Pennsylvania, Philadelphia, Pennsylvania, USA.; 2 Department of Clinical and Experimental Medicine, Unit of Infectious Diseases, University of Messina, Messina, Italy.; 3 Center for the Study of Biological Complexity, Virginia Commonwealth University, Virginia, USA.; 4 Department of Biomedical Engineering, University of Delaware, Newark, Delaware, USA.

**Keywords:** AIDS/HIV, T cells

## Abstract

Despite the efficacy of antiretroviral therapy (ART), HIV persists in a latent form and remains a hurdle to eradication. CD4^+^ T lymphocytes harbor the majority of the HIV reservoir, but the role of individual subsets remains unclear. CD4^+^ T cells were sorted into central, transitional, effector memory, and naive T cells. We measured HIV DNA and performed proviral sequencing of more than 1900 proviruses in 2 subjects at 2 and 9 years after ART initiation to estimate the contribution of each subset to the reservoir. Although our study was limited to 2 subjects, we obtained comparable findings with publicly available sequences. While the HIV integration levels were lower in naive compared with memory T cells, naive cells were a major contributor to the intact proviral reservoir. Notably, proviral sequences isolated from naive cells appeared to be unique, while those retrieved from effector memory cells were mainly clonal. The number of clones increased as cells differentiated from a naive to an effector memory phenotype, suggesting naive cells repopulate the effector memory reservoir as previously shown for central memory cells. Naive T cells contribute substantially to the intact HIV reservoir and represent a significant hurdle for HIV eradication.

## Abstract 2

Near full length sequencing of HIV DNA shows that naive CD4 T cells harbor a higher percentage of intact proviruses compared to other CD4 T subsets and represent one of the major contributors to the reservoir.

## Introduction

With the advent of antiretroviral therapy (ART), the natural history of HIV infection has dramatically changed. Viral loads typically decline to undetectable levels. This is followed by immune recovery, which leads to an improvement in quality of life and life span (1). Nevertheless, the virus persists as a latent reservoir, which is capable of viral rebound upon ART interruption (2).

In recent years, next-generation sequencing has become a powerful tool to study the proviral landscape over time (3–8). We reasoned that studying the proviral character deeply in different cellular subsets might provide insights into their contribution to HIV persistence. Our reasoning was largely based on a prior study from our group demonstrating differential selection against proviral sequences depending on their genetic makeup (6). In that study, we observed relative enrichment of proviruses with a 5′ deletion over time, consistent with refs. 9 and 10. These deletions resulted in uniform loss of the strong donor splice site D1 and preservation of the second strong donor splice site D4. We hypothesized that this enrichment may occur because 5′-deleted proviruses might be less efficient at HIV protein expression while retaining the ability to splice aberrantly to downstream genes, including genes involved in cell division/survival. Simultaneously, proviruses with a preserved D1 and an ORF were depleted over time, perhaps because encoded HIV proteins were expressed more efficiently (6).

Most efforts to study the HIV reservoir have focused on memory CD4^+^ T cells because this heterogeneous cellular subset carries the highest levels of HIV DNA in vivo (11–19). Moreover, C-C chemokine receptor type 5–tropic (CCR5-tropic) HIV virions are responsible for transmission (20), reinforcing the central role of memory T cells, since these cells express higher levels of CCR5 compared with naive CD4^+^ T cells (T_N_ cells) (21). Studies of naive infection have been limited, due to the perceived dominant role of memory T cells in HIV persistence. While several groups detected HIV DNA in T_N_ cells both in vitro and in vivo (12–19, 22–28), the levels of HIV DNA within T_N_ cells were generally 10-fold lower than in memory cells (11–19), suggesting that the HIV DNA found in T_N_ cells might be due to residual contamination of memory lymphocytes. Moreover, since T_N_ cells are more resistant to direct HIV infection by CCR5-tropic viruses in vitro (24), these cells were considered unlikely to play a central role in HIV pathogenesis. However, naive cells can be infected with C-X-C motif chemokine receptor 4–tropic (CXCR4-tropic) virus in vitro (24), as they express high levels of CXCR4. Given that HIV often evolves to express CXCR4-tropic envelope (29), there is potential for T_N_ cells to contribute to the reservoir. Moreover, T_N_ cells have unique characteristics that could lead to distinct hurdles to HIV eradication. These include longer intermitotic half-life than memory T cells (30), resistance to HIV expression after integration (24, 31–33), and the ability to give rise to all memory T cell subsets.

In the current study, we sought to study the selection pressures within and between cellular subsets using a longitudinal sequencing analysis approach. To address these questions, we sorted CD4 cellular subsets from 2 chronically infected individuals at 2 and 9 years after ART by adopting previously described sorting approaches (13, 16–18, 31, 33, 34) and obtained comparable findings in 3 other individuals through publicly available sequences (14). We performed near full-length (NFL) proviral sequencing of sorted cellular subsets and monitored dynamics of intact and defective proviruses. By tracking proviral composition over time, we uncovered evidence that infected T_N_ cells are a significant contributor to the reservoir with the potential to repopulate the memory reservoir.

## Results

### Sorting of CD4^+^ T cell subsets after 2 and 9 years of ART.

**Figure 1:**
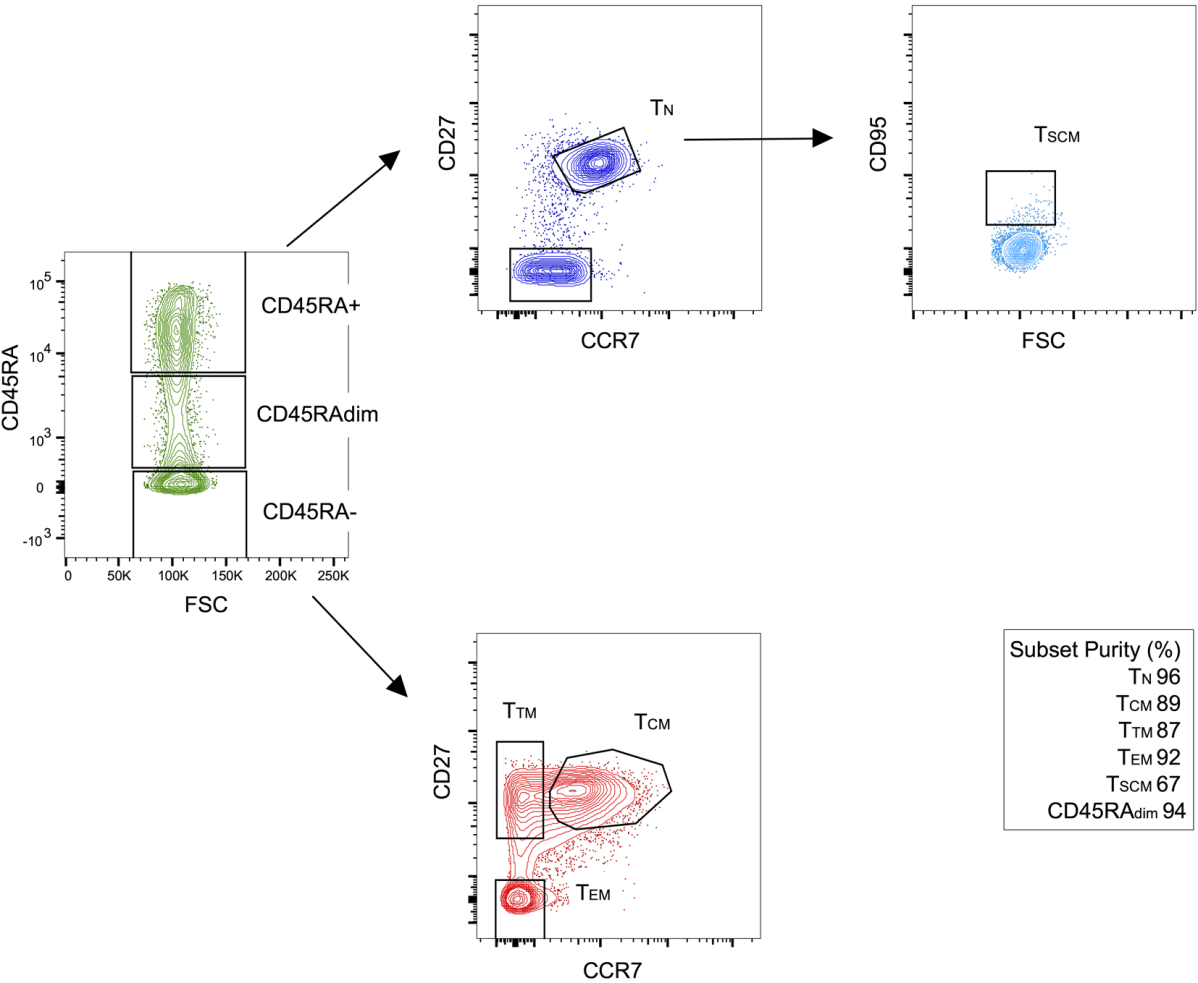
Sorting strategy to obtain cellular subsets. CD4^+^ T cells were negatively selected by immunomagnetic beads from peripheral blood mononuclear cells from 2 HIV-infected donors on ART at 2 time points corresponding to 2 and 9 years after ART initiation .T_N_ cells were enriched by sorting for CD45RA^+^CCR7^+^CD27^+^ cells. T_CM_ cells were CD45RA^–^CCR7^+^CD27^+^, T_TM_ cells were CD45RA^–^CCR7^–^CD27^+^, while T_EM_ cells were CD45RA^–^CCR7^–^CD27^–^. Finally, CD45RA_dim_ cells were also collected in an effort to sample almost all CD4^+^ T cell subsets. For selected experiments, we also sorted CD95^–^ T_N_ cells (CD45RA^+^CCR7^+^CD27^+^CD95^–^) and T_SCM_ cells (CD45RA^+^CCR7^+^CD27^+^CD95^+^). Purity was determined after sorting by flow cytometry. Here, we show 1 representative experiment. For these sorting experiments, we did not sort CD45RA^+^CD27^–^CCR7^–^ cells (T_EMRA_ cells; ref. 66). The low purity of T_SCM_ cells can be explained by the low levels of CD95 expressed by these cells, resulting in limited separation between the CD95^+^ and CD95^–^ population. T_EMRA_, effector memory reexpressing CD45RA.

To study reservoir dynamics in different cellular subsets, we chose to study 2 individuals in whom we had previously measured reservoir decay (6) (Figure 1 and Supplemental Tables 1 and 2; supplemental material available online with this article; https://doi.org/10.1172/jci.insight.133157DS1). Both subjects had undetectable plasma viral load at the time of the apheresis. We sorted CD3^+^CD8^–^ T cells by flow cytometry into T_N_ cells, central memory (T_CM_), transitional memory (T_TM_), effector memory (T_EM_) and CD45_dim_ cells as described in Figure 1, Table 1, Table 2, and Methods. We measured the amount of HIV DNA per million cells by quantitative PCR (qPCR). The percentage of T cells for each subset and the estimate of HIV DNA per million cells in each subset are reported in Table 1 (6). We also chose to sort CD45_dim_ cells, as this population made up a sizeable portion of T cells but was difficult to categorize as either naive or memory. We found CD45_dim_ cells also contained substantial levels of HIV DNA. The HIV DNA levels measured in each subset likely reflect the fraction of HIV that is integrated, since the enrolled individuals were on suppressive ART for more than 2 years at the time of apheresis (35, 36). Consistent with the literature (12–19), HIV DNA levels were lower in T_N_ cells compared with the combined memory population (Table 1). Given that memory T cells carry higher levels of HIV DNA than T_N_ cells, it was important to rule out that the few DNA sequences we identified in T_N_ cells might be due to contaminating memory cells. Based on postsorting purity, we estimated the fraction of proviral sequences that could be attributed to contaminating memory cells. Given that, on average, 4% of the sorted T_N_ cells were memory T cells, these contaminants could only account for a small fraction (less than 22%) of the HIV DNA sequences. Thus, the majority of the HIV DNA retrieved from T_N_ cells could not be attributed to contaminating cells with a memory phenotype. Of greater significance, sequence analysis revealed that the character and composition of proviruses in the naive subset was substantially different from those in the memory subsets.

### T_N_ cells are a significant contributor to the intact reservoir, despite lower levels of HIV DNA.

**Figure 2:**
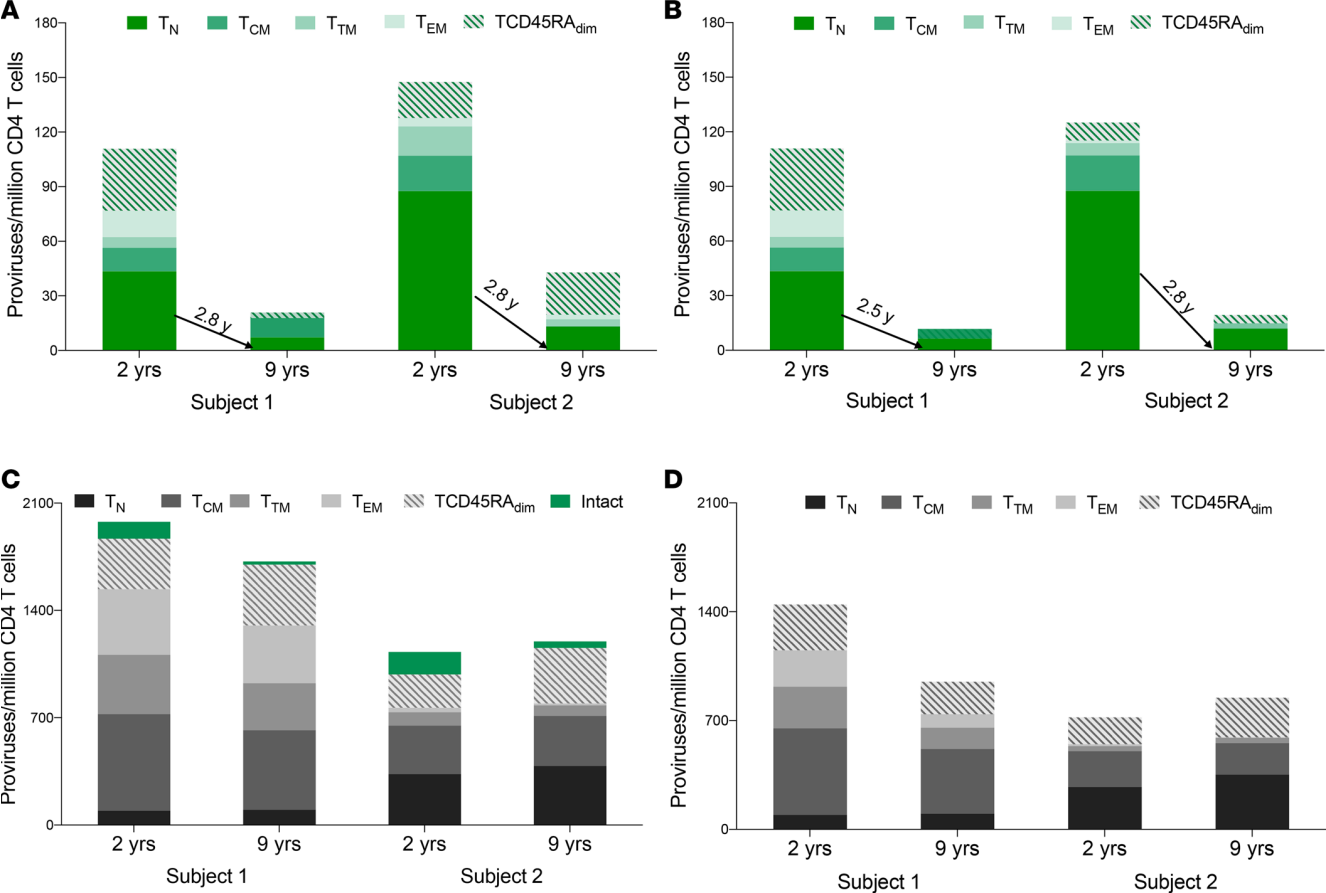
Contribution of T cell subsets to proviral DNA at 2 and 9 years after ART initiation. (**A** and **B**) Contribution of each T cell subset to the total number of intact proviruses at 2 time points before (**A**) and after removing large clonal populations (**B**). The half-life of intact HIV for T_N_ cells is shown in the graph. The half-life of each subset is provided in Supplemental Table 3. T_N_ cells represent a major contributor to the intact reservoir at both time points. Removal of repeated sequences revealed that large clones were more frequent among the memory subsets compared with the naive population. Notably, the half-life of intact proviruses in T_N_ cells was minimally affected by removing repeated sequences in contrast to the half-life of the memory cell subsets, which was shortened. (**C** and **D**) Contribution of each T cell subset to the total number of defective proviruses at 2 time points before (**C**) and after removing large clonal populations (**D**). We calculated the level of defective HIV DNA before and after removing repeated sequences at both time points. We calculated the percentage of defective proviruses by subtracting the percentage of intact proviral sequences (depicted as green bars in **C**) from the total number of HIV proviruses (as estimated by qPCR). This percentage was used to estimate the absolute number of defective proviruses. The levels of defective proviruses minimally changed over time in both individuals. Moreover, T_N_ cells contributed less to the pool of defective HIV in comparison with intact HIV.

To genetically characterize the HIV reservoir in sorted cellular subsets, we amplified and sequenced 942 full-length proviruses from Subject 1 and 975 proviruses from Subject 2 after sorting (Figure 1 and Table 2). We aligned the de novo assembled sequences to HXB2 and identified intact proviral sequences, as described in Methods and in ref. 6. To determine the number of intact proviruses contributed by each major T cell subset, we multiplied the total HIV DNA per subset (Table 1) by the fraction of intact proviruses within that subset (Table 1 and Figure 2). This provided an estimate of intact proviruses from each subset (Methods and Table 1). As an example, for Subject 1 at the first time point, we measured 655 HIV copies/million cells in T_N_ cells (Table 1, column A). We estimated the contribution of T_N_ cells to the total pool of CD4^+^ T cells by flow cytometry (21%, column C) and the fraction of proviruses that was intact by NFL proviral sequencing (32%, column B). We used these estimates to calculate the number of intact HIV per million naive CD4 cells (column D) as A × B × C (655 × 0.21 × 0.32 = 43 intact HIV/million naive CD4^+^ T cells). In both individuals, T_N_ cells were a major contributor (Figure 2, A and B). T_N_ cell contribution to the intact reservoir was 39% and 34% in Subject 1 and 59% and 31% in Subject 2 at the first and second time points, respectively. The intact reservoir decay in PBMCs (as estimated in ref. 6) and in T_N_ cells was similar, with a half-life between 2 and 3 years (Figure 2 and Supplemental Table 3). Notably, when we minimized the effects of large clones by removing repeated sequences, the contribution of the naive reservoir was more prominent at the later time points (Figure 2B). In contrast, total HIV DNA decayed minimally over time in both subjects (Figure 2C), which is consistent with the minimal change of defective proviral DNA between these 2 time points (Supplemental Table 3 and Figure 2, C and D). This is consistent with studies monitoring integrated HIV DNA showing a minimal proviral decline after starting ART in chronically infected individuals (36–38). In addition, the contribution of T_N_ cells to defective proviral DNA was minor when compared with the intact reservoir. This suggests that intact proviruses may have a survival advantage within T_N_ cells, possibly because of reduced HIV expression, which in turn might protect them from viral and/or immune cytotoxicity (39). Alternatively, intact proviruses could form more efficiently in T_N_ cells; however, a recent study suggests greater than 90% of proviruses are intact after a single-round infection of primary CD4^+^ T cells, making this hypothesis less likely (3).

### Proviral sequences in naive and effector memory cells have distinct characteristics that represent a continuum.

**Figure 3:**
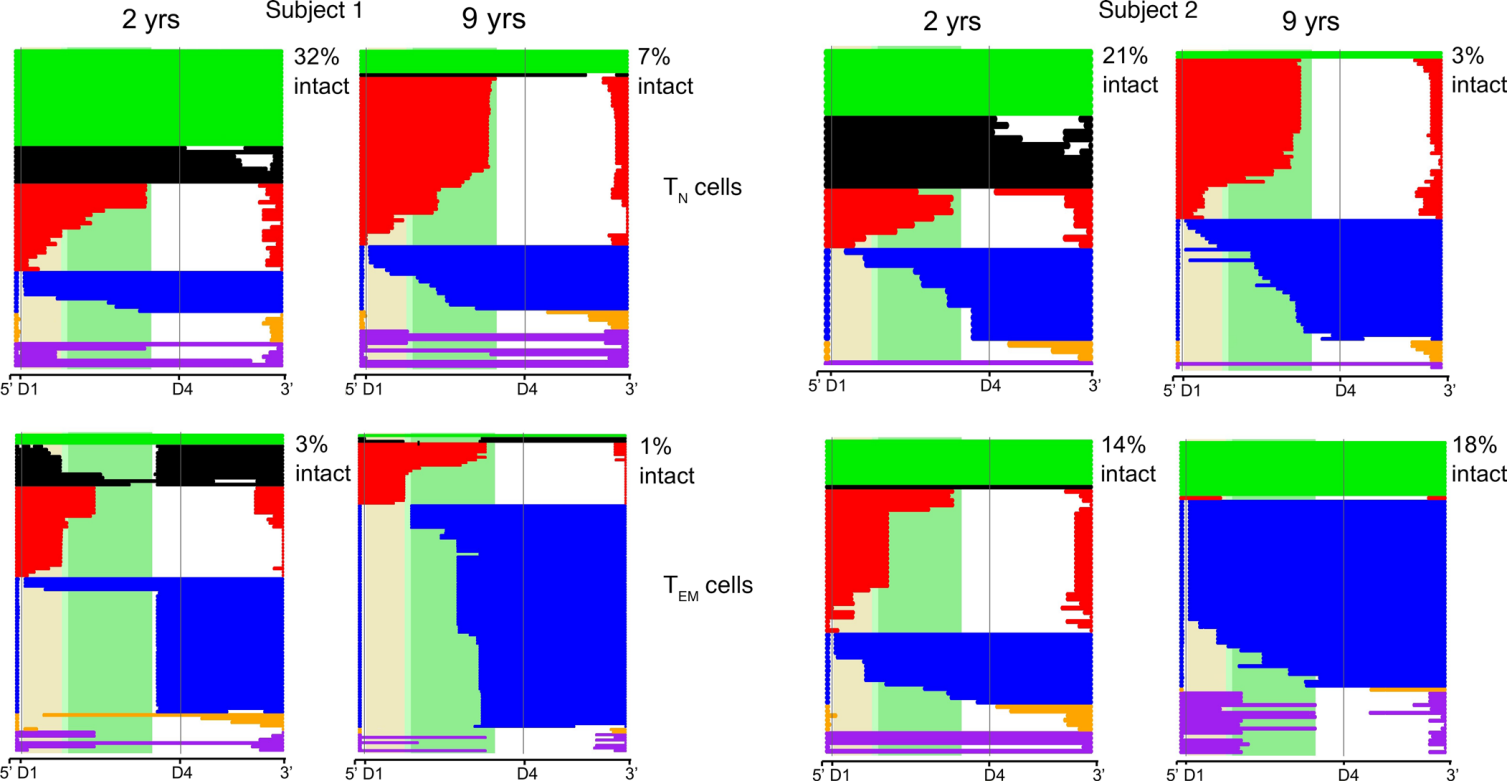
Deletion maps for proviruses retrieved from T_N_ and T_EM_ cells at 2 and 9 years after ART initiation. Intact and defective proviruses from T_N_ and T_EM_ cells were aligned to HXB2 at an early and late time point after ART initiation. Each horizontal bar represents 1 proviral sequence. Proviruses are color-coded based on size and location of deletion. Green bars are intact (D1^+^D4^+^), black bars are nearly intact (D1^+^D4^+^), red bars are 3′ deleted (D1^+^D4^–^), blue bars are 5′ deleted (D1^–^D4^+^), and yellow bars are massively deleted (D1^–^D4^–^) proviruses. Hypermutated proviruses are represented as purple bars. The shaded beige, light green, and dark green regions correspond to the gag, gag-pol, and pol regions of HXB2, respectively. The percentage of intact proviruses is reported in the upper right corner of each deletion map.

We wanted to explore the genetic pressures exerted on HIV in different cellular subsets by studying the composition of proviral DNA. We previously provided evidence that we could identify distinct categories of proviruses that are meaningful, as they undergo differential selection pressures (6). In Figure 3, we color coded proviral sequences into 6 categories based on their size and the location of deletions. These same categories could be defined by the presence or absence of D1 and D4 splice sites: intact (green, D1^+^D4^+^), nearly intact (black, D1^+^D4^+^), 3′ deleted (red, D1^+^D4^–^), 5′ deleted (blue, D1^–^D4^+^), massively deleted (yellow, D1^–^D4^–^), and hypermutated (purple, D1^+/–^D4^+/–^) proviruses (6). Each horizontal bar represents 1 assembled provirus aligned to HXB2. As expected, the majority of the proviruses were defective, and most had large deletions consistent with recent reports (4–6, 14).

T_EM_ cells showed the strongest evidence of selection, as they were dominated by distinct identical sequences at both time points. These identical proviral sequences detected more than once were likely to represent clones that we named “proviral clones.” This is likely a reasonable assumption, since most repeated sequences were defective proviruses and, therefore, not capable of spreading infection. For intact proviruses, it is possible that identical sequences might have different integration sites, since these intact proviruses have the capacity to release virus and cause viral spread. However, given that these individuals are on suppressive ART, this is only remotely possible.

Interestingly, there was a relative increase in blue proviral clones with 5′ deletions (D1^–^D4^+^ proviruses), suggesting a selective advantage for this category of proviruses consistent with refs. 6, 9, 10. In both subjects, there was a decline in the absolute level of D1^+^ proviruses with ORFs over time (green, black and red) again consistent with (6, 9). In Subject 2, we observed a relative increase in intact proviruses, but these proviruses were all identical proviral sequences at the second time point, suggesting clonal expansion. Overall, there was evidence of large turnover of proviral DNA in the T_EM_ subset.

The composition of the proviral DNA was strikingly different in the T_N_ subset compared with T_EM_ cells. Surprisingly, a large fraction of proviruses in the T_N_ compartment were intact or nearly intact. These D1^+^D4^+^ proviruses declined over time and appeared to be unique sequences, as they were only detected once. In contrast, defective proviruses within the T_N_ compartment represented a minor fraction, and their character did not change over time, consistent with the idea that these defective proviruses were under less selective pressure in T_N_ cells in comparison with T_EM_ cells. To summarize, intact and nearly intact (D1^+^D4^+^) defective proviruses declined more rapidly in the T_N_ subset relative to other defective proviral categories. In contrast, in the T_EM_ subset, there was negative selection against some categories of defective proviruses (D1^+^ORF^+^) and relative positive selection for others (D1^–^D4^+^), resulting in the presence of distinct large clones and a high turnover rate. Thus, the genetic composition and longitudinal changes observed in the T_N_ and T_EM_ reservoir were distinct (see also Supplemental Tables 4 and 5). Deletion maps for T_CM_ and T_TM_ cells are provided in Supplemental Figure 1 and show intermediate phenotypes between the extremes of T_N_ and T_EM_ cells.

### Large proviral clones are more frequent in T_EM_ compared with T_N_ cells.

We aligned all the HIV sequences to compare the extent of repeated proviral sequences in T_N_ and T_EM_ cells. We identified 2 repeated sequences out of 212 total sequences (<1%) for T_N_ cells and 133 repeated sequences out of 209 (64%) for T_EM_ cells in Subject 1. Subject 2 followed the same pattern, with 10% repeated sequences within the T_N_ compartment and 71% repeated sequences among T_EM_ cells. The fractions of detectable proviral clones was significantly higher in T_EM_ versus T_N_ cells at both time points in each subject by a 2-tailed χ^2^ test (*P* < 0.001). Moreover, the number of clones was significantly greater at the second time point in T_EM_ cells (*P* < 0.05) but not in T_N_ cells. Taken together, these results suggest that T_N_ and T_EM_ cells represent 2 ends of a continuum, with a much higher frequency of proviral clones as cells differentiate from a naive to an effector memory phenotype (Supplemental Table 4).

### CD95^–^ T_N_ and T_SCM_ cells contain a similar fraction of intact proviruses.

**Figure 4:**
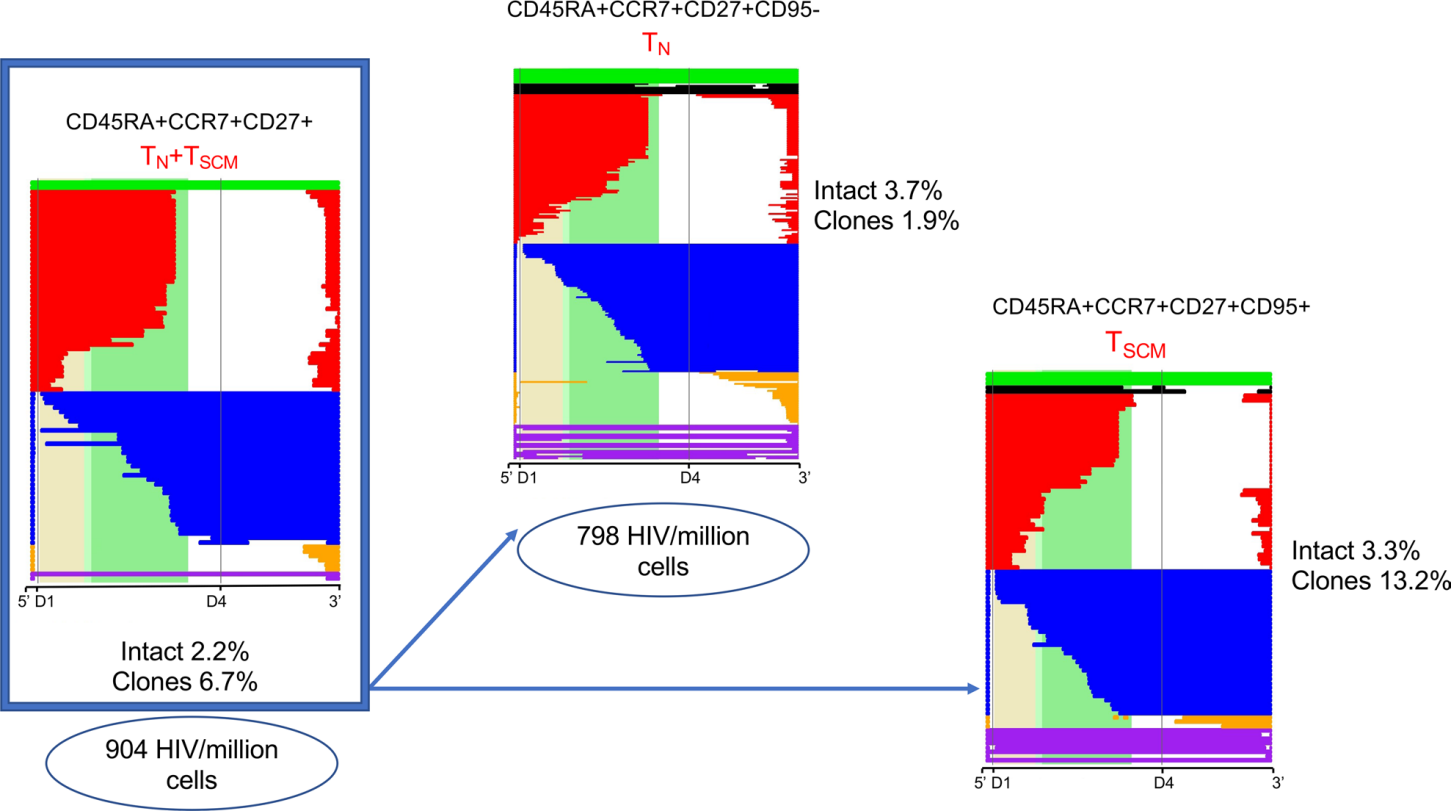
Comparison of HIV DNA levels and reservoir composition by NFL sequencing in T_N_ versus T_SCM_ cells for Subject 2. The sorting experiment described in Figure 1 was repeated, separating T_SCM_ from CD95^–^ T_N_ cells. CD95^–^ T_N_ cells were defined as CD45RA^+^CCR7^+^CD27^+^CD95^–^ cells, while T_SCM_ cells were defined as CD45RA^+^CCR7^+^CD27^+^CD95^+^ cells. Sorted cells were used to measure HIV DNA levels by qPCR and to obtain proviral sequences. These proviral sequences were used to generate deletion maps, as described in Figure 3. T_SCM_ and CD95^–^ T_N_ cells had similar levels of intact HIV, with a higher fraction of clones in T_SCM_ cells.

**Figure 5:**
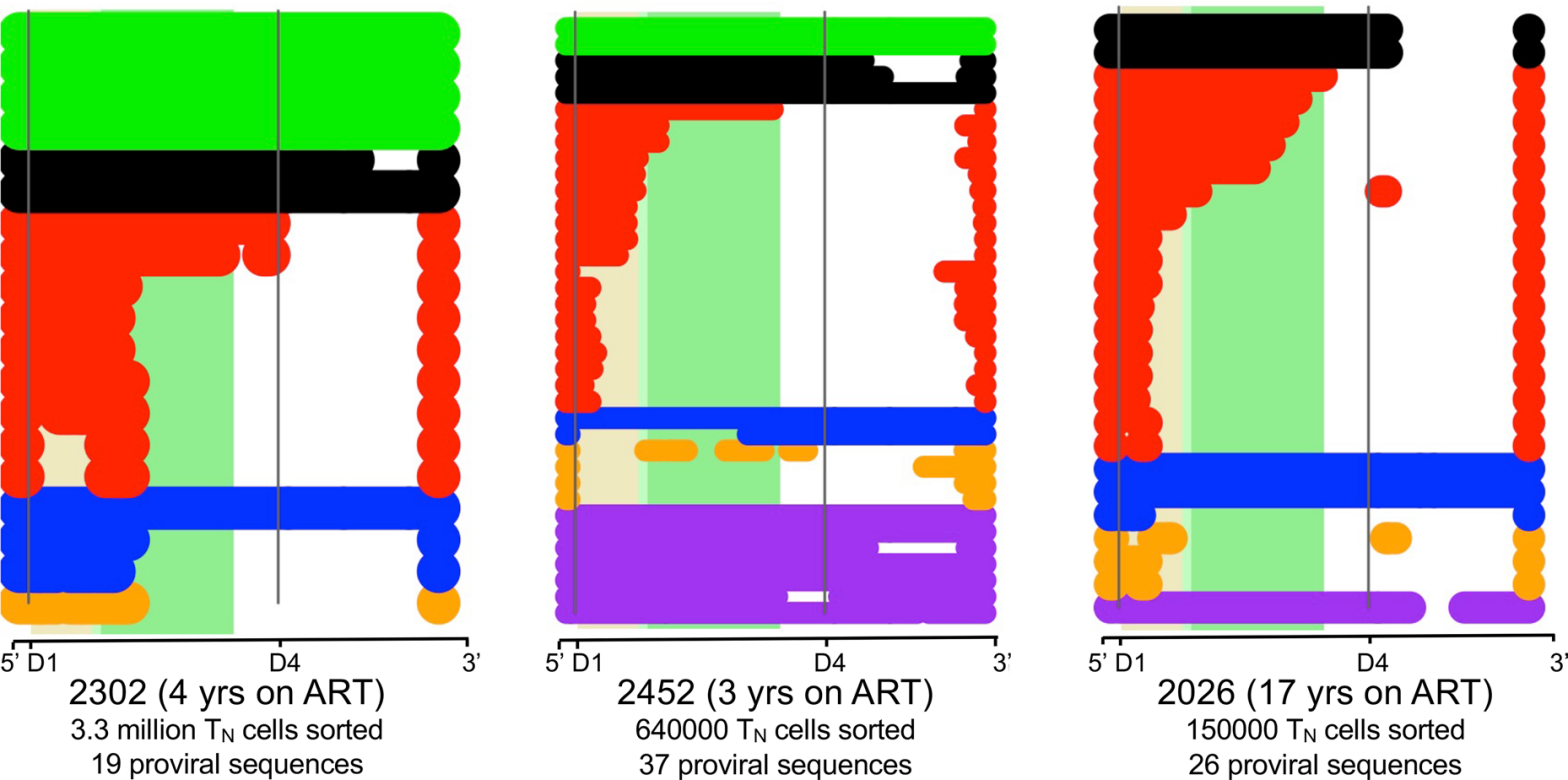
Sequences from sorted naive cells support that T_N_ cells contribute to the HIV reservoir. Sequences from sorted T_N_ cells were downloaded from Hiener et al. (14) and analyzed using our pipeline. The deletion maps were generated with the same bioinformatic tools used in Figure 3 and Figure 4 and Supplemental Figures 1 and 2 for our 2 subjects. No intact proviruses were detected for subject 2026, which might be explained by the low number of sorted T_N_ cells, as well as the long time the patient has been on ART. Nonetheless, the composition of the reservoir in T_N_ cells in these 3 individuals closely matches the proviral composition detected in T_N_ cells in Figure 3.

The experiments presented thus far were based on a sorted CD45RA^+^CCR7^+^CD27^+^ T_N_ population. This population contains 0.2%–6% of stem-cell like memory (T_SCM_) cells, which have been reported to harbor latent HIV (15). In order to assess the contribution of T_SCM_ cells to our results, we sorted CD45RA^+^CCR7^+^CD27^+^CD95- cells (CD95- T_N_) and CD45RA^+^CCR7^+^CD27^+^CD95^+^ (T_SCM_) cells at the second time point, for which we had banked a sufficiently large number of cells. We measured the amount of HIV DNA in CD95^–^ T_N_ cells for both individuals by qPCR. For Subject 1, we estimated 411 HIV copies/million cells in CD95^–^ T_N_ versus 468 in T_N_ cells. In Subject 2, we measured 798 copies/million cells in CD95^–^ T_N_ versus 904 copies/million cells in T_N_ cells. This suggests that CD95^–^ T_N_ and T_SCM_ cells contain similar levels of HIV DNA. For Subject 2, we amplified and sequenced proviruses from both the CD95^–^ T_N_ and T_SCM_ compartment, obtaining 216 and 91 proviruses, respectively. For Subject 1, we were able to obtain proviruses only from CD95^–^ T_N_ cells (53 sequences), since T_SCM_ cells were extremely rare (<0.001% of the total CD4^+^ T cell population) (Supplemental Figure 2). In both individuals, the percentage of intact proviruses was similar to the one observed in T_N_ cells. Finally, deletion maps did not reveal differences in the character of the proviral sequences amplified from CD95^–^ T_N_ and T_N_ cells except that CD95^–^ T_N_ cells had fewer detectable proviral clones (Figure 4). Taken together, these results suggest that the HIV DNA detected in T_N_ cells is not due to contaminating T_SCM_ cells. While our in-depth sequencing analysis was limited to 2 individuals after 2 and 9 years of ART, Figure 5 shows a similar infection frequency and proviral character of T_N_ cells from 3 additional HIV-infected individuals on ART enrolled in ref. 14. Thus, we were able to confirm our findings of an important role for T_N_ cells in HIV persistence by applying our pipeline analysis to published data from Hiener et al. (14).

### Analysis of coreceptor tropism suggests that T_N_ cells harbor CCR5-tropic infection.

**Figure 6:**
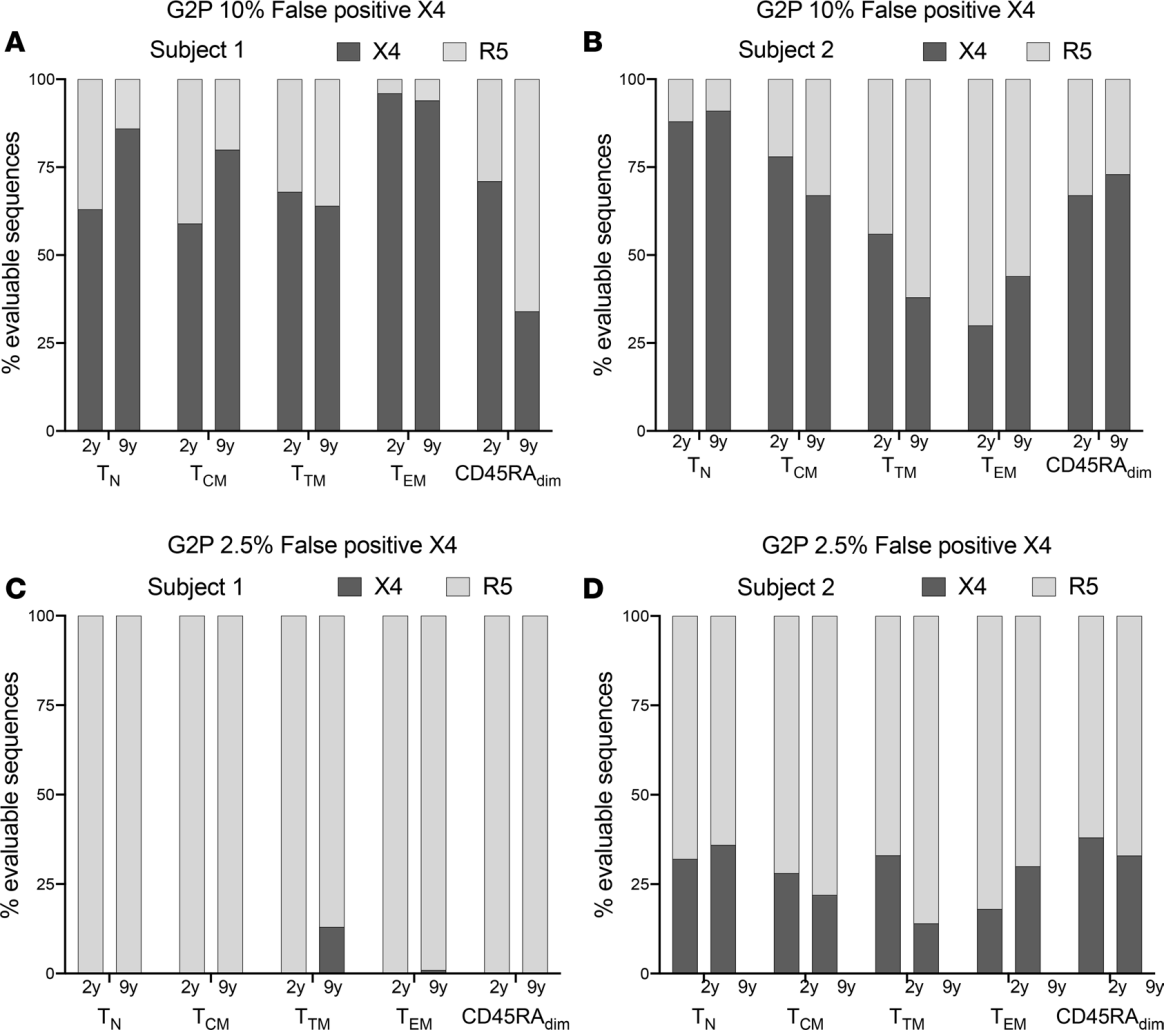
Analysis of coreceptor tropism using Geno2Pheno (G2P) suggests that T_N_ cells might harbor CCR5-tropic HIV. We used G2P to study coreceptor tropism of proviral sequences retrieved from different subsets. We included all the evaluable env sequences. (**A** and **B**) We used G2P with a 10% FPR, where the FPR indicates the likelihood to misclassify a provirus as CXCR4-tropic (which is based on phenotypic assays). When using G2P FPR 10%, both subjects showed a predominance of CXCR4-tropic sequences. (**C** and **D**) We used G2P with a 2.5% FPR. With G2P FPR 2.5%, most evaluable env ORFs were predicted to be CCR5-tropic for Subject 1 at both time points (**C**), while Subject 2 had, on average, 30% CXCR4-tropic sequences across subsets (**D**). Interestingly, with either 10% or 2.5% FPR, T_N_ cells were predicted to contain CCR5-tropic sequences in both individuals.

We were next interested to determine if T_N_ cells would only harbor CXCR4-tropic viruses, given that these cells appear to be resistant to CCR5-tropic HIV in vitro (24). We analyzed all proviruses with an intact env ORF using 2 bioinformatic tools: WebPSSM (position-specific scoring matrices) and Geno2Pheno (G2P) (40, 41). Both programs use algorithms based on critical amino acids in the V3 loops. The literature has reported limitations of both prediction tools (42, 43). Underlying this limitation is the fact that a significant fraction of HIV proviruses is dual tropic (44–49). Recognizing these limitations, we chose to analyze the proviral sequences with both a high and low false-positive rate (FPR). When analyzing sequences with the high FPR setting, we are likely overrepresenting CXCR4-tropic proviruses. We decided that this would be a reasonable approach to provide a lower-bound estimate of the frequency of CCR5-tropic proviruses within T_N_ cells. With the most restrictive criteria for classifying proviruses as CCR5-tropic (i.e., G2P 10% FPR), in both individuals, a sizeable fraction of T_N_ cells was predicted to be infected with CCR5-tropic HIV (35% and 14% for Subject 1 at the first and second time point, and 12% and 9% for Subject 2; Figure 6, A and B). While more investigation is needed to address these results with phenotypic studies, our findings suggest that T_N_ cells can harbor CCR5-tropic strains.

### UpSet plots identify relationships between cellular subsets.

We studied the relationship between cellular subsets by identifying repeated sequences (proviral clones) in different cellular subsets. These repeated sequences were used to create modified UpSet plots. The circles connected by lines represent proviral clones that were grouped based on 2 criteria: the time point and the cellular subset from which they were detected. This modified UpSet analysis revealed that different subsets often harbored the same proviral clones, suggesting that the differentiation pathway of T cells from a naive to an effector memory phenotype could play a role in HIV persistence.

**Figure 7:**
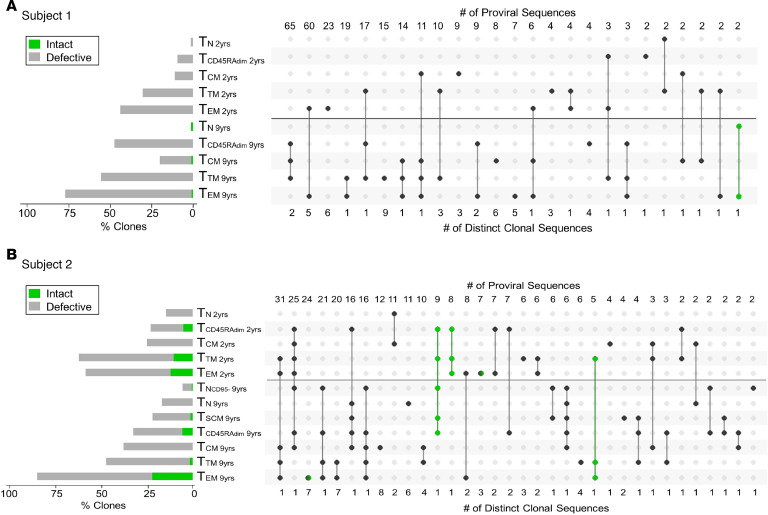
Modified proviral clone UpSet plots. (**A** and **B**) Modified proviral clone UpSet plot for Subject 1 (**A**) and Subject 2 (**B**) shows that clonal expansion progressively increases with cell differentiation and is more prominent among defective proviruses. Proviral clones were identified as repeated sequences. The horizontal bars on the left side show the percentage of repeated sequences found in each subset (green for intact sequences and gray for defective ones). A black horizontal line separates the 2 time points (2 and 9 years after ART initiation). Intact proviral clones are shown in green. For those proviral clones that could be detected in multiple subsets, a solid line was used to connect these subsets. The numbers at the top of the UpSet plot represent the number of proviral sequences that could be found in the same subsets within a category. The numbers below the UpSet plot represent the number of distinct clonal sequences. For example, for Subject 2 (**B**), we identified 308 repeated sequences. The first column shows that we detected 1 distinct clone made up by 31 proviral sequences in T_EM_ and T_TM_ at both time points, as well as T_CM_ cells at the second time point. For Subject 1, we identified 303 repeated sequences (**A**). We also identified 5 intact clones for Subject 2 (**B**) and 1 proviral clone for Subject 1 (**A**). yrs, years after ART initiation.

The horizontal bars on the left of the UpSet capture the percentage of proviral clones in each subset at each time point (gray for defective clones and green for intact ones). The number of repeated proviral sequences detected is provided at the top of the UpSet plot, while the number of distinct sequences is shown below the UpSet plot. Proviral clones depicted in green are intact. As an example, for Subject 1, we detected 2 repeated intact proviral sequences in T_EM_ and T_N_ cells representing 1 distinct clonal sequence (Figure 7A, last column in green). As an additional example, for Subject 2 (Figure 7B), column 1 shows that we detected 31 repeated proviral sequences representing 1 distinct clone. This distinct proviral sequence was detected within T_EM_ and T_TM_ cells at both time points and T_CM_ cells at the second time point. Column 3 shows that we identified 7 distinct sequences in T_EM_ cells at the second time point (for a total number of 24 repeated proviral sequences) and one of them happened to be intact (as indicated by the hemi-green circle). Overall, we identified 5 intact clones for Subject 2 and 1 intact proviral clone for Subject 1, as also shown in the phylogenetic tree in Supplemental Figure 3. The limited number of intact proviral clones suggests that selection against intact proviruses is stronger than against defective ones due to either direct cytotoxicity or immune pressure. Nonetheless, while selection against intact HIV is strong, T_N_ cells containing intact proviruses appear to repopulate the more differentiated cellular subsets.

Our data are consistent with previous reports showing an increase in the proportion of proviral clones over time (6, 50). A sizeable fraction of proviral clones persisted and were detected at both time points 7 years apart from each other. The proportion of proviral clones in each subset progressed from T_N_ to T_EM_ cells (Supplemental Table 4, Figure 7, and Supplemental Figure 3). This follows the expected pathway of cellular differentiation with T_N_ cells dividing less frequently than T_SCM_ < T_CM_ < T_TM_ < T_EM_ cells (30, 51). How CD45RA_dim_ cells fit into this pathway is unclear, as these cells have not been routinely studied. In our study, we found that CD45RA_dim_ cells have a higher number of clones than T_N_ (and often T_CM_) cells but fewer than T_TM_ or T_EM_ cells, suggesting that they might represent a population in transition from a naive to a memory phenotype and vice versa.

## Discussion

The importance of T_N_ cell infection has generally been discounted as inconsequential, as the low level of infection found in this cellular subset have been attributed to contamination of sorted T_N_ cells by memory or stem-cell memory T cells and to reversion of memory cells to a naive phenotype. Our study challenges these explanations by suggesting a central role for T_N_ cells in HIV persistence.

We first show that naive infection cannot be due to contaminating memory cells by examining HIV DNA levels (Figure 1 and Table 1). We further support the case for naive infection with the surprising finding that these cells significantly contribute to the intact reservoir by proviral sequencing (Figure 2), as they represent one of the largest contributing subsets. We then provide genetic evidence that the character of the proviruses retrieved from infected T_N_ cells is distinct from any other subset (Figure 3 and Figure 4) with fewer identical sequences. The distinct nature of this subset further argues against the idea that infection could be due to contamination by or reversion from memory T cells. When we depleted T_SCM_ cells from the T_N_ cells, we found the intact reservoir persisted with fewer clonal species. Thus, the infection detected in T_N_ cells cannot be due to these rare cells (Figure 4). We used our bioinformatic pipeline to show similar findings in publicly available databases (Figure 5). Finally, we focused our analysis on the clonal populations present in CD4^+^ T cells, showing a steady progression of clone size with T cell maturation (Figure 7).

### T_N_ cells may repopulate the memory reservoir.

Our evidence that T_N_ cells may give rise to other subsets includes the observation that identical sequences are shared between T_N_ cells and other subsets. Moreover, the number of proviral clones increases as cells become more differentiated (Figure 7 and Supplemental Table 4). Our analysis shows a steady progression in the number of clonal sequences from T_N_ to T_SCM_ to T_CM_ to T_TM_ to T_EM_ cells. Each clonal population likely arose from a single progenitor infection event. Most of the intact proviruses in T_EM_ cells appear to be large expanded clones. Given that we sampled a very small fraction of the total proviral population (<0.00001%), detection of a proviral sequence more than twice in any sample suggests this clone made up a substantial portion of all proviruses in the blood. By contrast, the intact reservoir in the T_N_ compartment appears to have fewer large proviral clones. Thus, a larger fraction of the intact proviruses detected in T_N_ cells likely represents separate successful infection events. From our data, we infer that infection events leading to the formation of an intact reservoir in T_N_ cells might be more common than previously recognized. Indeed, our evidence that clonal progeny arose from less differentiated subsets suggests that many of the expanded intact clones in the more differentiated memory subsets started as T_N_ or T_CM_ infections. This overarching finding of a progression from more unique to more clonal sequences as we move from naive to more differentiated memory cells is consistent with Lee et al. (8).

### T_N_ cells provide a unique hurdle to HIV persistence.

T_N_ cells have the longest half-life (51, 52) among cellular subsets. Evidence is accumulating that T_N_ cells express less HIV RNA and proteins in vitro and in vivo (31–33, 53). Moreover, these cells have been suggested to be more resistant to cytotoxic T lymphocyte (CTL) killing (39). These features could result in less negative selection pressure against infected T_N_ cells in comparison with memory T cells, which might explain, in turn, why the intact reservoir appears to be more protected in naive cells. It remains to be established whether T_N_ cells are more resistant to latency reversal, as only a few studies have investigated T_N_ infection (24) and fewer still have addressed latency reversal in cellular subsets (54, 55).

### HIV may play a role in proviral clonal expansion in T_EM_ cells.

Notably, D1^–^D4^+^ (5′deleted blue) proviruses in T_EM_ cells expanded relative to all other proviral categories and were largely clonal. At first glance, this might appear counterintuitive, as we would expect enrichment for massively deleted proviruses (yellow, D1^–^D4^–^) over other categories, since these massively deleted proviruses have a lower potential to express HIV proteins and, therefore, should be subject to limited negative selection. However, massively deleted D1^–^D4^–^ proviruses were not the most persistent proviral category. This strongly suggests that the enrichment for D1^–^D4^+^ proviruses we observed over time might be due to positive selection. We have hypothesized that positive selection might result from aberrant splicing of these D1^–^D4^+^ proviruses to downstream oncogenes, which, in certain scenarios, could lead to increased cellular proliferation (6, 56). D1 and D4 are the strongest donor splice sequences in HIV. Given the organization of the HIV genome, with only 1 weak splice site acceptor after D4 but many splice acceptor sequences after D1, it appears reasonable that D4 would often splice aberrantly to downstream genes.

### HIV DNA levels in cellular subsets obscure important selection pressures.

Consistent with other reports (12–19), our work shows that measures of total HIV DNA provide a misleading picture of the reservoir size, since T_N_ cells have the lowest integration levels (12–16, 19). To estimate the real contribution by each subset, it is essential to distinguish intact and defective proviruses. Recently, Zerbato et al. showed similar levels of HIV in T_N_ and T_CM_ cells by viral outgrowth, even though T_CM_ cells had higher levels of integrated HIV DNA (17). This study indirectly corroborates our finding of a higher percentage of intact proviruses in T_N_ cells.

### Unique differences in reservoir character and selection pressures in T_N_ cells were revealed by NFL proviral sequencing.

It can be argued that we should further divide T_N_ cells into subsets based on additional markers. Historically, T_N_ cells were defined using the same markers we used in our study (13, 16–18, 31, 34). More importantly, even without using additional markers, there were clear differences in the proviral character of different sorted subsets, suggesting that our approach identified meaningful functional differences. While the CD45RA^+^CCR7^+^CD27^+^CD95^–^ subset contains cells that are not truly naive from a functional perspective, the ability of this subset to recirculate (due to the presence of CCR7), their lack of recent activation (as suggested by the presence of CD45RA), and their longer half-life compared with memory T cells (51) provide a rationale for grouping these cells into one population. The limited number of proviral clones retrieved from naive cells is consistent with the properties of this population that divides rarely and recirculates continuously between the blood and lymphoid tissues.

When we analyzed the CD45RA^+^CCR7^+^CD27^+^CD95^–^ naive population, we noticed similar integrated and intact HIV levels in comparison with the T_SCM_ compartment (CD45RA^+^CCR7^+^CD27^+^CD95^+^). This would suggest that, at least in these 2 individuals, T_SCM_ cells do not differ greatly in character from CD95^–^ T_N_ cells, except for the higher frequency of repeated proviral sequences observed in T_SCM_ cells. Again, our findings are consistent with Zerbato et al., showing no significant differences in total HIV DNA and replication-competent HIV in the total T_N_ population (containing T_SCM_ cells) in comparison with the CD95^–^ T_N_ compartment (17).

It might seem surprising to observe CCR5-tropic sequences in T_N_ cells (Figure 6) since these cells are highly resistant to infection with CCR5-tropic HIV in vitro. In fact, we were unable to detect infection after in vitro inoculation of CD4^+^ T cells (ref. 24 and O’Doherty, unpublished observations). It is possible that CCR5-tropic HIV can infect naive cells inefficiently due to minimal or transient CCR5 expression on T_N_ cells (57, 58), as CCR5 can be transiently upregulated by various stimuli (24, 59, 60). Moreover, lack of detection of CCR5 by flow cytometry would not prove its absence on the cellular surface, since flow cytometry has a detection limit of 50–100 molecules and some databases report very low levels of CCR5 RNA (61). Alternately, T_N_ cells may upregulate CCR5 after direct contact with antigen presenting cells in lymph nodes, since they can recirculate. Consistent with this hypothesis, it has recently been reported that T_N_ cells in lymph nodes harbor a high percentage of replication-competent HIV (62). In addition, T_N_ infection with a CCR5-tropic virus appears to occur in the presence of CCL19 and B cells (63). Memory cells harboring HIV may revert to a naive phenotype after being infected with a CCR5-tropic virus (64). It should also be noted that our analysis of coreceptor tropism is based on genotypic predictions, not phenotypic tests. As a consequence, we cannot rule out dual tropism conferred by determinants located outside of the V3 loop region, as previously described by others (44–49). While beyond the scope of this manuscript, further studies are needed to improve our understanding of the role of coreceptor tropism in T_N_ infection.

A major limitation of our study is the restricted number of individuals we enrolled. We made the deliberate decision to sequence a large number of proviruses per cellular subset rather than to sequence fewer proviruses in many subjects. Our study complements the results published by other groups that sorted cellular subsets in a larger number of HIV-infected individuals — but with fewer sequences per patient and fewer sequences per cellular subset (4, 5, 8, 14). To address this limitation, we applied our bioinformatic pipeline to analyze sequences obtained after sorting T_N_ cells from a recently published study (ref. 14 and Figure 5). While this analysis suggests our findings may apply more broadly, it also demonstrates the need for deep sequencing within each subset to provide robust conclusions.

In conclusion, our work suggests that the contribution of T_N_ cells to HIV persistence should be reevaluated. T_N_ cells may be more resistant to immune clearance and viral cytotoxicity due to their lower viral expression as reported by refs. 31–33 and 53. Thus, the T_N_ reservoir may be a formidable hurdle to HIV cure. Moreover, infected T_N_ cells can give rise to infected memory T cells through differentiation and, thereby, have the potential to continuously repopulate the memory subset, obscuring the true decay rate of infected memory cells. Further studies to explore how to perturb latency in different cellular subsets are needed.

## Methods

### Patients and samples.

We used samples from 2 HIV-1–infected individuals whose clinical history and reservoir decay curves were previously described (6). Apheresis samples were collected at 2 and 9 years after ART initiation. Before ART initiation, Subject 1 experienced a slow CD4^+^ T cell decline and a nadir of 295 CD4 cells/μL after 21 years of infection. Subject 2 had a more rapid progression, with a nadir of 0 CD4 cells/μL after 6 years of infection (Supplemental Tables 1 and 2). Both subjects had < 20 copies of HIV RNA/mL at the time of the apheresis.

### Sorting and purity for qPCR and NFL proviral sequencing.

CD3^+^CD8^–^ T lymphocytes were negatively selected from peripheral blood mononuclear cells (PBMCs) by an immunomagnetic bead–based protocol (Stem Cell Technologies, catalog 19052).

Purified T lymphocytes were then sorted by fluorescence activated cell sorting (BD FACS Aria III sorter) into the following subsets: CD3^+^CD8^–^ T_N_ (CD45RA^+^CCR7^+^CD27^+^), CD95^–^ T_N_ (CD45RA^+^CCR7^+^CD27^+^CD95^–^), T_SCM_ (CD45RA^+^CCR7^+^CD27^+^CD95^+^), T_CM_ (CD45RA^–^CCR7^+^CD27^+^), T_TM_ (CD45RA^–^CCR7^–^CD27^+^), T_EM_ (CD45RA^–^CCR7^–^CD27^–^), and CD45RA_dim_ cells (Figure 1) using PE-Cy7 anti-CD27 (clone 0323, Invitrogen), BV421 anti-CD45RA (clone 5H9, BD Biosciences), BB700 anti-CCR7 (clone 3D12, BD Biosciences), and APC anti-CD95 (clone DX2, BD Biosciences). The following filters were used: 450/50 Violet for BV421, 780/40 Green for PeCy7, 710/50 Blue for CCR7, and 660/20 for APC (BD Biosciences). FlowJo v10.6 software was used for analysis.

### DNA quantification and NFL proviral sequencing.

Genomic DNA was purified using the Gentra Puregene kit (QIAGEN), and HIV-DNA was measured by HIV LTR qPCR (6) with several dilutions and replicates (between 9 and 15 replicates per sample). Proviruses were then amplified using NFL PCR at limiting dilution and sequenced on an Illumina MiniSeq as described in ref. 6. An in-house HIV genome analysis program developed in R studio was used to identify intact proviruses (6). The criteria to define a provirus as intact included the presence of 9 ORFs without premature stop codon and insertions/deletions, the presence of 3–4 stem loops at the psi packaging site, as well as the presence of the critical donor and acceptor splice sequences and the Rev response element (6).

### Analysis of proviral clones and classification of proviral sequences based on coreceptor usage.

Sequences were aligned to HXB2 to identify deletions using Multiple Alignment with Fast Fourier Transformation (MAFFT) (65). Proviruses with 100% sequence identity were considered clones. Identical sequences (defined as proviral clones) were grouped into clonal families. To increase the power of our analysis and detect more proviral clones, we used our complete database, including the proviral sequences retrieved from PBMCs published in ref. 6.

Proviral coreceptors were classified by analyzing the V3 region of the HIV envelope protein gp120 using WebPSSM tool (40) and G2P (41). For G2P, we performed our analysis using a 10% and 2.5% FPR. Since the WebPSSM and G2P 2.5% FPR gave comparable results, we decided to only show results from the G2P analysis in Figure 6.

### Calculation of the contribution of cellular subsets to the HIV reservoir.

Thirty-two to 304 proviral sequences were available per time point to estimate the percentage of intact proviruses. The contribution of intact proviruses to the reservoir by each subset (C_s_) was calculated with the following formula: (intact proviruses/1 × 10^6^ subset cells) × (subset cells/1 × 10^6^ CD4^+^) = Cs.

The absolute number of intact proviruses contributed per subset was calculated as: intact proviruses = total HIV LTR DNA per subset cells × % intact in that subset.

The percent contribution of each subset was calculated as: (C_s_/CT_N_ + CCD45RA_dim_ + CT_CM_ + CT_TM_ + CT_EM_) × 100, where CT_N_ indicates contribution T_N_ cells, CCD45RA_dim_ indicates contribution CD45RA_dim_ cells, CT_CM_ indicates contribution T_CM_ cells, CT_TM_ indicates contribution T_TM_ cells, and CT_EM_ indicates contribution T_EM_ cells.

### Statistics.

A 2-tailed χ^2^ test was used to compare the fraction of proviral clones in T_EM_ versus T_N_ cells in each individual, as well as the number of clones within the same subset at 2 time points. *P* < 0.05 was considered significant.

For data presented in Supplemental Table 3, confidence intervals were computed as exact posteriors, using log-normal variance computed from repeated measures for qPCR measurements and binomial statistics for intact versus defective ratios. R and Excel software were used for statistical analyses. Prism GraphPad was used to generate the figures presented in the paper.

### Study approval.

The study was approved by the IRB at the NIH and at the University of Pennsylvania, and written informed consent was received from participants before inclusion in the study.

## Author contributions

MRP, EVR, and UO designed the study. EVR, MRP, and MC conducted the experiments. EVR, MRP, LC, SW, RZ, GN, and UO analyzed the data. UO wrote the manuscript with significant contributions from MRP, as well as additional contributions from RZ, SW, and EVR. RZ provided statistical support for data analysis. All the authors read and approved the final version of the manuscript.

## Supplementary Material

Supplemental data

## Figures and Tables

**Table 1:**
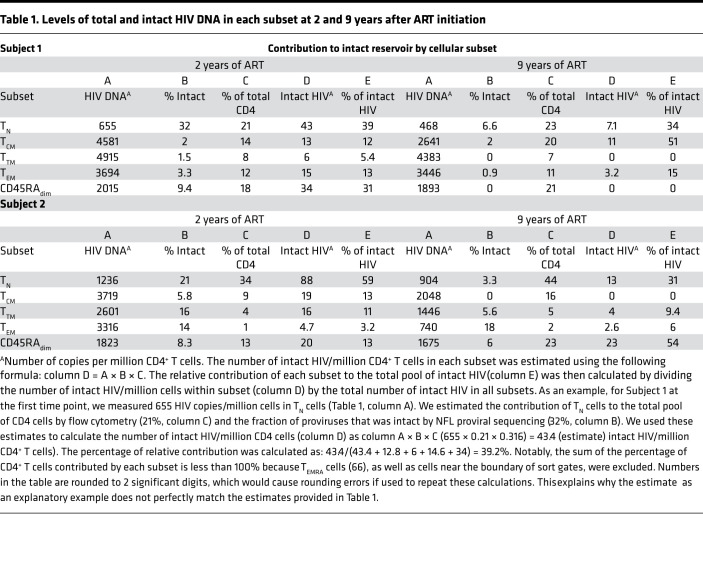
Levels of total and intact HIV DNA in each subset at 2 and 9 years after ART initiation

**Table 2:**
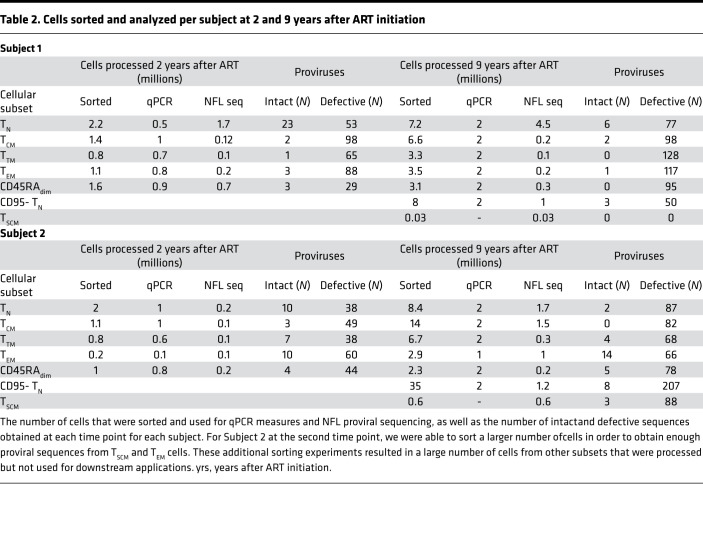
Cells sorted and analyzed per subject at 2 and 9 years after ART initiation

## Version 1 10/15/2020

Electronic publication
