# Supplementary material for: Persistence of an intact HIV reservoir in phenotypically naive T cells
Source: JCI Insight. 2020 Oct 15;5(20):e133157. doi: 10.1172/jci.insight.133157 (PMC7605525; doi:10.1172/jci.insight.133157)
Supplement: Supplemental data [file jciinsight-5-133157-s012.pdf]

# Persistence of an intact HIV reservoir in phenotypically naive T cells

Emmanuele Venanzi Rullo*,[1,2] Marilia Rita Pinzone*,[1] LaMont Cannon*,[1,3] Sam Weissman,[1] Manuela Ceccarelli,[1,2] Ryan Zurakowski,[4] Giuseppe Nunnari,[2] Una O'Doherty[1]

* These authors contributed equally

## Supplementary Data

### Table S1. Clinical characteristics of the subjects enrolled in the study

| Subject | Age* | Sex | Year of infection | CD4 T-cell nadir (cells/μl (year)) | Viral load zenith (copies/ml (year)) |
|---|---|---|---|---|---|
| **1** | 47 | M | 1984 | 295 (1998) | 114,422 (1998) |
| **2** | 36 | M | 1993 | 0 (1999) | 225,000 (2001) |

*Age at the time of first apheresis collection

Abbreviations: M, man

### Table S2. Virological and immunological parameters at two time points after ART initiation in the subjects enrolled in the study

| Subject | Year | Time on ART (years) | ART | Viral load (copies/ml) | CD4 T-cells (cells/μl) |
|---|---|---|---|---|---|
| **1** | 2008 | 1.9 | ATV/r TDF/FTC | <50 | 617 |
| | 2015 | 8.2 | ATV/r TDF/FTC | <20 | 718 |
| **2** | 2007 | 2.1 | ATV/r 3TC D4T | <50 | 629 |
| | 2015 | 9.9 | ATV/r 3TC RAL | <40 | 768 |

Abbreviations: ABC, abacavir; ART, antiretroviral therapy; ATV, atazanavir; D4T, stavudine; r, ritonavir; RAL, raltegravir; TDF/FTC, tenofovir/emtricitabine; 3TC, lamivudine.

| | Subject 1 (Half-lives with 95% C.I., years) | | | |
|---|---|---|---|---|
| | Intact | Intact (repeated sequences removed) | Defective | Defective (repeated sequences removed) |
| $T_N$ | 2.8 (1.8-5.5) | 2.5 (1.6-4.8) | (9.4-Inf) | (9.7-Inf) |
| $T_{CM}$ | (2.5-Inf) | (1.7-Inf) | 25 (13-273) | 17 (9.7-62) |
| $T_{TM}$ | (0.9-Inf) | (0.9-Inf) | (11-Inf) | 7.3 (4.8-16) |
| $T_{EM}$ | (1.5-Inf) | (0.9-Inf) | (14-Inf) | 5.2 (3.4-10) |
| $CD45RA_{dim}$ | 1.8 (0.8-7.8) | 1.8 (0.8-7.7) | (30-Inf)* | (6.4-Inf)* |
| | Subject 2 (Half-lives with 95% C.I., years) | | | |
| | Intact | Intact (repeated sequences removed) | Defective | Defective (repeated sequences removed) |
| $T_N$ | 2.8 (1.6-6.6) | 2.8 (1.6-6.6) | (68-Inf)* | (47-Inf)* |
| $T_{CM}$ | 2.6 (1.0-89) | 2.6 (1.0-88) | (34-Inf)* | (13-Inf) |
| $T_{TM}$ | (2.3-Inf) | (2.6-Inf) | (7.4-Inf)* | (10-Inf)* |
| $T_{EM}$ | (3.8-Inf) | 2.1 (0.9-10) | 6.4 (4.4-12) | 3.3 (2.3-5.4) |
| $CD45RA_{dim}$ | (3.7-Inf) | (1.8-Inf) | (27-Inf)* | (14-Inf) |

*Median fit is actually growth, not decay

**Table S3. Half-life of intact and defective proviruses in cellular subsets before and after removing repeated sequences.**

The median decay rate is reported only for those subsets with a statistically significant decay rate.

Data are reported in years (95% confidence interval (C.I.)). Confidence intervals were computed as exact posteriors, using log-normal variance computed from repeated measures for qPCR measurements and Binomial statistics for intact vs. defective ratios as in (1)

(1) Luo R, Piovoso MJ, Zurakowski R. Modeling uncertainty in single-copy assays for HIV. *J Clin Microbiol*. 2012;50(10):3381-3382. doi:10.1128/JCM.01254-12

| | Subject 1: 2 years | | | | | | Subject 2: 2 years | | | | | | |
|---|---|---|---|---|---|---|---|---|---|---|---|---|---|
| | $T_N$ | $T_{CM}$ | $T_{TM}$ | $T_{EM}$ | CD45RA$_{dim}$ | | $T_N$ | $T_{CM}$ | $T_{TM}$ | $T_{EM}$ | CD45RA$_{dim}$ | | |
| Proviral sequences (N) | 76 | 100 | 66 | 91 | 32 | | 48 | 52 | 45 | 70 | 48 | | |
| Unique sequences (N) | 75 | 89 | 46 | 51 | 29 | | 41 | 39 | 17 | 29 | 37 | | |
| % repeated sequences | 1.3 | 11 | 30 | 44 | 13 | | 15 | 25 | 62 | 59 | 23 | | |
| Intact proviral sequences (N) | 23 | 2 | 1 | 3 | 3 | | 10 | 3 | 7 | 10 | 4 | | |
| Repeated sequences among intact proviruses (N) | 0 | 0 | 0 | 0 | 0 | | 0 | 0 | 4 | 6 | 2 | | |
| | Subject 1: 9 years | | | | | | Subject 2: 9 years | | | | | | |
| | $T_N$ | $T_{CM}$ | $T_{TM}$ | $T_{EM}$ | CD45RA$_{dim}$ | CD95-$T_N$ | $T_N$ | $T_{CM}$ | $T_{TM}$ | $T_{EM}$ | CD45RA$_{dim}$ | CD95-$T_N$ | $T_{SCM}$ |
| Proviral sequences (N) | 83 | 100 | 128 | 118 | 95 | 53 | 89 | 82 | 72 | 80 | 83 | 215 | 91 |
| Unique sequences (N) | 82 | 80 | 57 | 27 | 45 | 53 | 74 | 51 | 38 | 12 | 56 | 203 | 71 |
| % clones | 1.2 | 20 | 55 | 79 | 53 | 0 | 17 | 38 | 47 | 85 | 33 | 5.6 | 22 |
| Intact proviral sequences (N) | 6 | 2 | 0 | 1 | 0 | 3 | 2 | 0 | 4 | 14 | 5 | 8 | 3 |
| Repeated sequences among intact proviruses (N) | 1 | 0 | 0 | 1 | 0 | | 0 | 0 | 1 | 14 | 4 | 1 | 1 |

**Table S4. Number of proviruses sequenced in cellular subsets at two time points and percentage of repeated sequences among total and intact proviruses**

Abbreviations: $T_N$: Naive CD4 T cells; $T_{CM}$: Central memory CD4 T cells; $T_{TM}$: Transitional memory CD4 T cells; $T_{EM}$: Effector memory CD4 T cells; $T_{SCM}$: Stem-cell like memory CD4 T cells; yrs: years after ART initiation.

| | Subject 1 | | | | | |
|---|---|---|---|---|---|---|
| | | Deleted | | | | |
| | Intact | D1+D4+ | D1+D4- | D1-D4+ | D1-D4- | Hypermutated |
| $T_N$ 2 yrs | 23 (30%) | 9 (12%) | 21 (28%) | 10 (13%) | 7 (9%) | 6 (8%) |
| $T_{CM}$ 2 yrs | 2 (2%) | 1 (1%) | 58 (58%) | 15 (15%) | 12 (12%) | 12 (12%) |
| $T_{TM}$ 2 yrs | 1 (2%) | 1 (2%) | 37 (56%) | 15 (23%) | 4 (6%) | 8 (12%) |
| $T_{EM}$ 2 yrs | 3 (3%) | 12 (13%) | 26 (29%) | 39 (43%) | 5 (5%) | 6 (7%) |
| $CD45RA_{dim}$ 2 yrs | 3 (9%) | 1 (3%) | 15 (47%) | 9 (28%) | 3 (9%) | 1 (3%) |
| $T_N$ 9 yrs | 6 (7%) | 1 (2%) | 44 (53%) | 17 (20%) | 5 (6%) | 10 (12%) |
| $T_{CM}$ 9 yrs | 2 (2%) | 0 (0%) | 48 (48%) | 28 (28%) | 14 (14%) | 8 (8%) |
| $T_{TM}$ 9 yrs | 0 (0%) | 5 (4%) | 39 (30%) | 60 (47%) | 17 (13%) | 7 (5%) |
| $T_{EM}$ 9 yrs | 1 (1%) | 2 (2%) | 23 (19%) | 83 (70%) | 2 (2%) | 7 (6%) |
| $CD45RA_{dim}$ 9 yrs | 0 (0%) | 0 (0%) | 29 (31%) | 58 (61%) | 4 (4%) | 4 (4%) |
| CD95- $T_N$ 9 yrs | 3 (6%) | 0 (0%) | 27 (51%) | 13 (25%) | 6 (11%) | 4 (8%) |

| | **Subject 2** | | | | | |
|---|---|---|---|---|---|---|
| | | **Deleted** | | | | |
| | **Intact** | **D1+D4+** | **D1+D4-** | **D1-D4+** | **D1-D4-** | **Hypermutated** |
| $T_N$ 2 yrs | 10 (21%) | 11 (23%) | 9 (19%) | 14 (29%) | 3 (6%) | 1 (2%) |
| $T_{CM}$ 2 yrs | 3 (6%) | 0 (0%) | 24 (46%) | 13 (25%) | 8 (15%) | 4 (8%) |
| $T_{TM}$ 2 yrs | 7 (16%) | 0 (0%) | 15 (33%) | 8 (18%) | 12 (27%) | 3 (7%) |
| $T_{EM}$ 2 yrs | 10 (14%) | 1 (1%) | 32 (46%) | 16 (23%) | 6 (9%) | 5 (7%) |
| $CD45RA_{dim}$ 2 yrs | 4 (8%) | 1 (2%) | 23 (48%) | 16 (34%) | 3 (6%) | 1 (2%) |
| $T_N$ 9 yrs | 2 (2%) | 0 (0%) | 45 (51%) | 34 (38%) | 6 (7%) | 2 (2%) |
| $T_{CM}$ 9 yrs | 0 (0%) | 0 (0%) | 33 (40%) | 20 (24%) | 16 (20%) | 13 (16%) |
| $T_{TM}$ 9 yrs | 4 (6%) | 2 (3%) | 15 (21%) | 35 (49%) | 6 (8%) | 10 (14%) |
| $T_{EM}$ 9 yrs | 14 (18%) | 0 (0%) | 1 (1%) | 48 (60%) | 1 (1%) | 16 (20%) |
| $CD45RA_{dim}$ 9 yrs | 5 (6%) | 1 (1%) | 18 (22%) | 41 (49%) | 12 (14%) | 6 (7%) |
| CD95- $T_N$ 9 yrs | 8 (4%) | 6 (3%) | 83 (38%) | 71 (33%) | 28 (13%) | 19 (9%) |
| $T_{SCM}$ 9 yrs | 3 (3%) | 2 (2%) | 41 (45%) | 34 (37%) | 3 (3%) | 8 (9%) |

## Table S5. Number of proviruses sequenced at two time points based on proviral categories

Abbreviations: $T_N$: Naive CD4 T cells; $T_{CM}$: Central memory CD4 T cells; $T_{TM}$: Transitional memory CD4 T cells; $T_{EM}$: Effector memory CD4 T cells; $T_{SCM}$: Stem-cell like memory CD4 T cells; yrs: years after ART initiation.

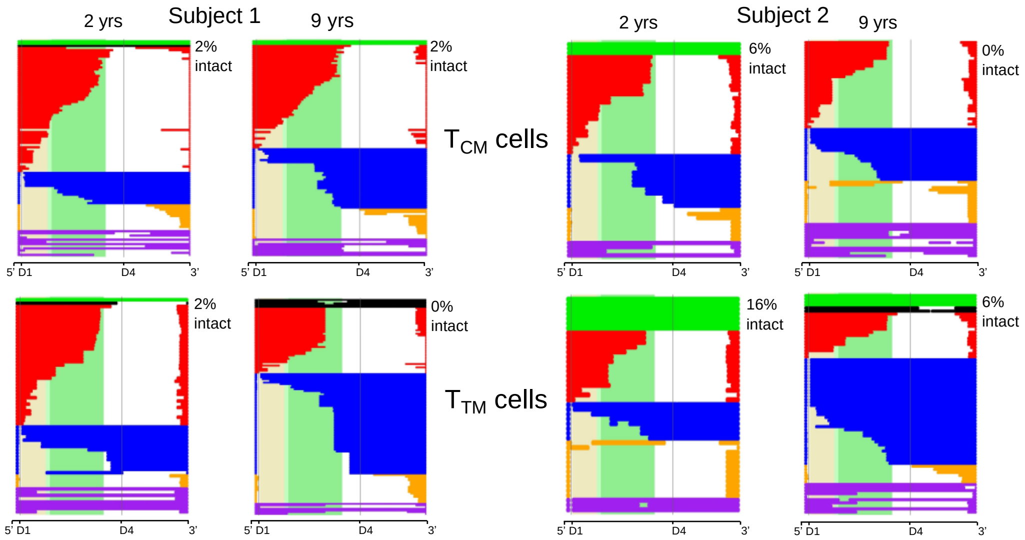


**Figure S1. Deletion map analysis of $T_{CM}$ and $T_{TM}$ cells reveals a genetic make-up in between $T_N$ and $T_{EM}$ cells.** DNA was purified and sequenced from sorted $T_{CM}$ and $T_{TM}$ cells. The sequenced amplicons were then analyzed using our bioinformatic pipeline. Intact and defective proviruses from $T_{CM}$ and $T_{TM}$ cells were aligned to HXB2 at an early and late time point. Green proviruses are intact (D1+D4+), black proviruses are nearly intact (D1+D4+), red proviruses are 3' deleted (D1+D4−), blue proviruses are 5' deleted (D1−D4+), and yellow proviruses are massively deleted (D1−D4-). Hypermutated proviruses are represented in purple. The shaded beige, light green, and dark green regions correspond to the gag, gag-pol, and pol regions of HXB2, respectively. The percentage of intact proviruses is reported in the upper right corner of each deletion map.

Abbreviations: $T_{CM}$: central memory CD4 T cells; $T_{TM}$: transitional memory CD4 T cells; yrs: years after ART initiation.

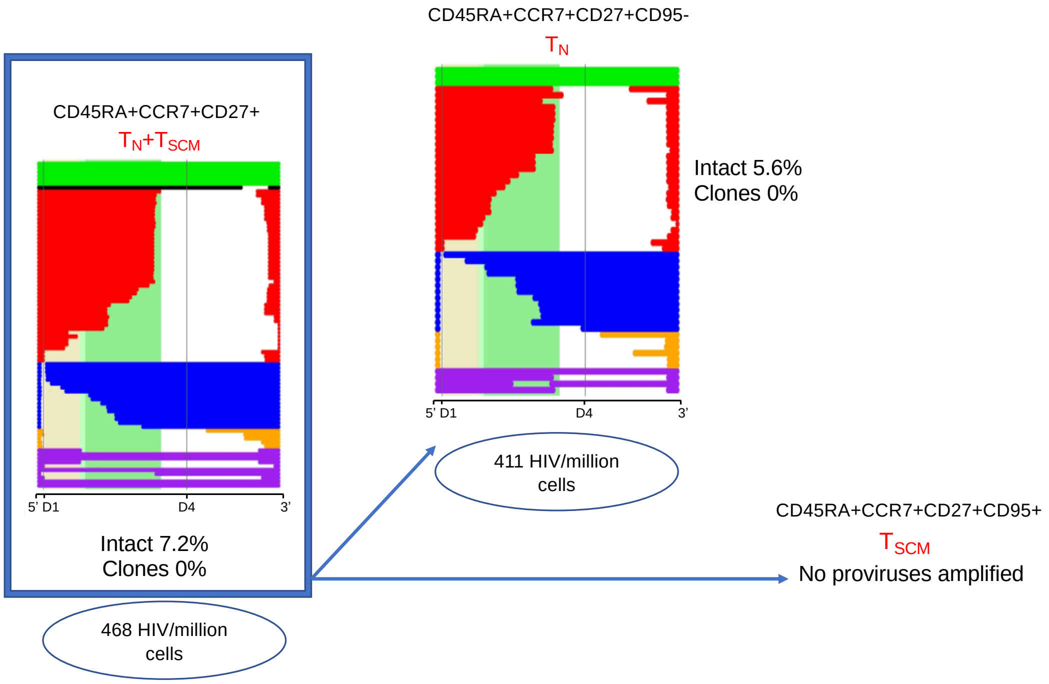


**Figure S2. Deletion map of $T_N$ *vs.* $T_{SCM}$ cells for Subject 1**. The sort experiment described in Figure 1 was repeated separating $T_{SCM}$ from CD95-$T_N$ cells. CD95-$T_N$ cells were defined as CD45RA+CCR7+CD27+CD95- while $T_{SCM}$ cells were CD45RA+CCR7+CD27+CD95+. Proviral DNA could not be sequenced from $T_{SCM}$ cells due to the extremely low levels of sorted cells. Proviral sequences retrieved from CD95-$T_N$ cells showed a similar percentage of intact HIV compared to CD45RA+CCR7+CD27+ $T_N$ cells.

Abbreviations: $T_N$, Naive CD4 T cells; $T_{CM}$, Central memory CD4 cells; $T_{TM}$, Transitional memory CD4 T cells; $T_{EM}$, Effector memory CD4 T cells; $T_{SCM}$, Stem-cell like memory T cells.

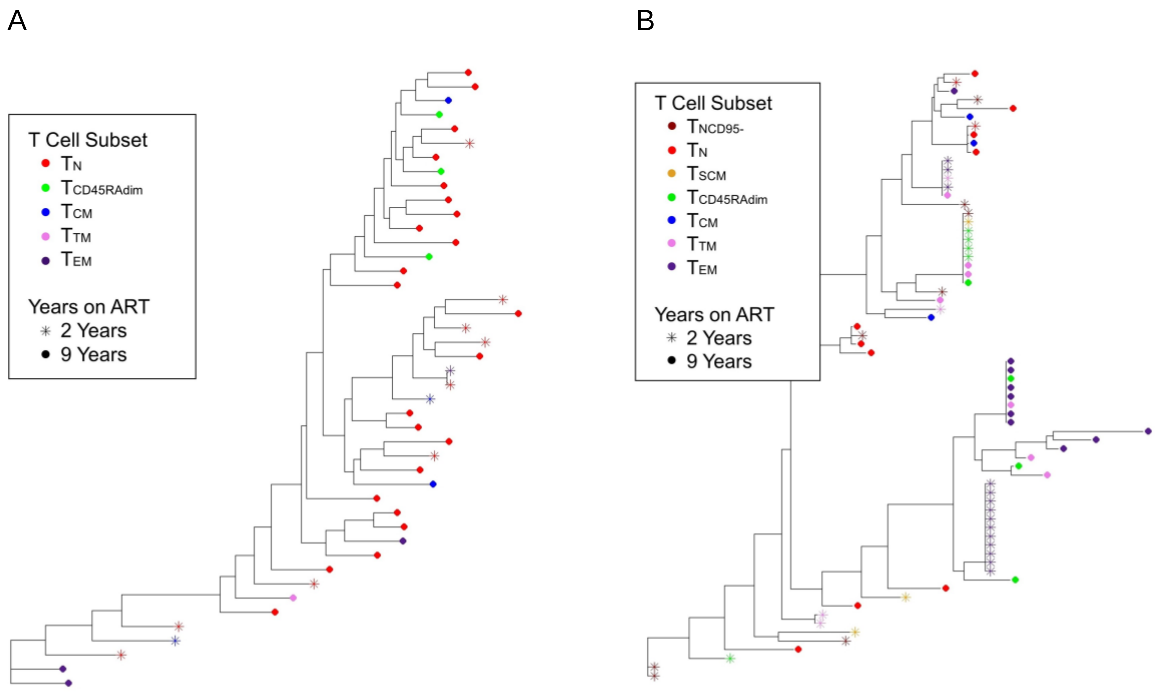


**Figure S3. Phylogenetic tree of intact proviruses for Subject 1 (panel A) and Subject 2 (panel B).** The phylogenetic tree included 44 intact proviral sequences for Subject 1 and 70 sequences for Subject 2. For Subject 1, we identified a proviral clone in $T_N$ and $T_{EM}$ cells at the second time point. For Subject 2, 4 clones were identified. For both subjects, identical intact proviral sequences were found among $T_N$ cells and more differentiated subsets, suggesting the potential for naive cells to repopulate the memory reservoir.

Abbreviations: $T_N$, Naive CD4 T cells; $T_{CM}$, Central memory CD4 cells; $T_{TM}$, Transitional memory CD4 T cells; $T_{EM}$, Effector memory CD4 T cells; $T_{SCM}$, Stem-cell like memory T cells.
